# Quantitative study on the three-dimensional defense of Puzhuang Suo-Fort ancient wall and the moat

**DOI:** 10.1371/journal.pone.0282537

**Published:** 2023-03-02

**Authors:** Shenge Shen, Lifeng Tan, Cheng Wang, Pengfei Zhao, Huanjie Liu, Jiayin Zhou, Yinggang Wang, Hong Yuan

**Affiliations:** 1 Department of Architecture, Tianjin University, Tianjin, China; 2 Department of Architecture, Tianjin Chengjian University, Tianjin, China; 3 Department of Architecture, Shandong Jianzhu University, Jinan, Shandong, China; Second Institute of Oceanography Ministry of Natural Resources, CHINA

## Abstract

The spatial layout of the coastal forts defense system of the Ming Dynasty of China has been studied in a relatively comprehensive way. Nonetheless, ancient defense mechanisms have not been fully revealed. Previous studies have focused more on the macro and meso levels. Studies into its microscopic construction mechanism need to be enhanced. This research attempts to quantify and validate the rationality of the ancient microscopic defense mechanism, using the ancient defense mechanism of Pu Zhuang Suo-Fort in Zhejiang Province as an instance. This study concentrates on the distribution of firepower strength beyond the walls of coastal defense forts, as well as the effect of wall height on firepower defense capabilities. There is a specific firepower attenuation area near the walls due to the firing blind area in the coastal forts defense system. And the construction of the moat plays an additive role in its defensive capability. Meanwhile, the height of the fort wall will also affect the range of the firing blind zone under Yangmacheng. In theory, there is a reasonable height range of the wall and a proper position of the moat. This height range can meet both good economy and defensive capabilities. In turn, the position of the moats and the height of the walls can verify the rationality of the construction mechanism of the coastal forts’ defense system.

## Introduction

Ancient Chinese construction and navigation technology began to develop early. Some scholars have studied the fortifications in ancient China based on Chinese historical books and records. According to historian R. D. Sawyer, ancient Chinese fortifications may have exerted a significant societal influence. He stated that the defensive solidity provided by the earliest walls and moats allowed for the progressive accumulation of products created by the weaving and handicraft industries, aided animal domestication, safeguarded the growth and dissemination of agriculture, and housed metallurgical workshops. By isolating the community from the outside environment, it also promoted social cohesiveness and a sense of identity [1]. Meanwhile, he has disclosed certain ancient Chinese strategies for attacking and defending, such as the significance of deep and wide moats plus solid and thick walls [2]. Historian Mark Cartwright has introduced the structure, mechanism and background of various Chinese fortifications in an article entitled Fortifications in Ancient Chinese Warfare [3]. Professor Gideon Shelach-Lavi and his team have used modern techniques such as intensive archaeological survey, GIS analysis, drone photography, and the analysis of satellite imagery to explore the function of the ancient Chinese wall in north-eastern Mongolia and the logic behind its construction [4]. Wu Zuobin, a scholar, mentioned that square city corners are easier to form a crossfire network for invaders. He mentioned the potential discrepancies in firepower distribution on various types of city walls [5].

The fortification wall’s horizontal and vertical firepower distribution has yet to be simulated. This paper reveals these microscopic laws. Meanwhile, the examination of the city wall’s defense strategy is primarily qualitative. Based on the understanding that the defense performance of higher city walls and wider moats is better, whether the numerical selection of the height and width has a quantifiable law is the primary concern of this paper. Various types of fortifications were the carriers of the defensive nature of military settlements. Massive earth walls with towers and encircling ditches or moats became most cities’ standard defense strategy [3]. The construction of traditional Chinese settlements reflects a certain degree of defensiveness. Since the study of military settlements in the Ming Dynasty was carried out, the study of fortifications has been involved. It is vital to conduct microscopic studies of engineering fortifications in the context of specific construction techniques and layout features under the study of Ming Dynasty military settlements.

The basic strategy of national defense during the Ming Dynasty was to defend. The Wei and Suo forts system was also born, combined with the politics and economic situation [6]. Zhejiang is located on the southeast coast of China. As early as the Tang and Song Dynasties, the maritime trade of Zhejiang was very developed [7]. Zhejiang’s strategic position and booming economy rendered it one of the areas most susceptible to invasion during the Ming Dynasty. The study group has effectively investigated Zhejiang’s coastal defense zone from macroscopic to mesoscopic levels in terms of site selection basis and layout features in current history [8]. The research of microscopic construction characteristics and basis needs to be strengthened.

Outside the wall, there was regularly a moat [9]. Invaders needed to cross the moat first if they wanted to break the fort. Moats were substantial barriers against the enemy. Some were formed based on natural water systems, while others were created by artificial digging [10]. And a suspension bridge was set outside the fort wall. If the Ming army needed to go in and out, they would put down the suspension bridge. If the invaders invaded, they would hang the suspension bridge [11]. The space between fort walls and moats was also the key to keeping out the enemy. Yangmacheng was usually set here to provide a barrier and combat field for the Ming army to ambush the invaders beneath the fort. When the enemy crossed the river, the Ming army would attack them under the fort behind Yangmacheng and on the fort wall [12]. Finally, a barbican was set outside the fort gate to resist the invaders who broke through the siege and arrived at the fort gates [13].

The wall height, distance between forts and Yangmacheng, and width of the manually excavated moat are discovered to be non-fixed values throughout the investigation and reference. As a result, it is hypothesized that their microscopic construction characteristics and building scale could be correlated to the defense mechanism in some manner.

This study takes Puzhuang Suo-Fort, a relatively well-preserved Suo-Fort in Zhejiang Province, as an example. Point cloud models are obtained through field research and modeled to restore and stimulate the walled space. Software such as GIS is used to quantify and analyze to reveal its combat mechanism. In turn, it verifies the science of constructing coastal defense fortifications at the microscopic scale.

## Research aims

This paper aims to study the firepower strength distribution outside the forts and the influence of fort wall heights on defense ability.

Taking the defense construction system of Puzhuang Suo-Fort as an example, this research analyzes it in two dimensions. To begin, in the horizontal direction, the strength of the firepower within a 100m space of the fort is measured and depicted in order to establish the total fort wall structure’s firepower distribution law Furthermore, outside the wall, determine the science of the position and magnitude of the moat and Yangmacheng. Moreover, in the vertical direction, investigate the quantitative relation between wall height and firing blind area. The most appropriate construction height range is established based on the economic considerations of wall construction, followed by the definition of the construction principle of the defense system.

## Material and methods

### Background analysis

**Fig 1:**
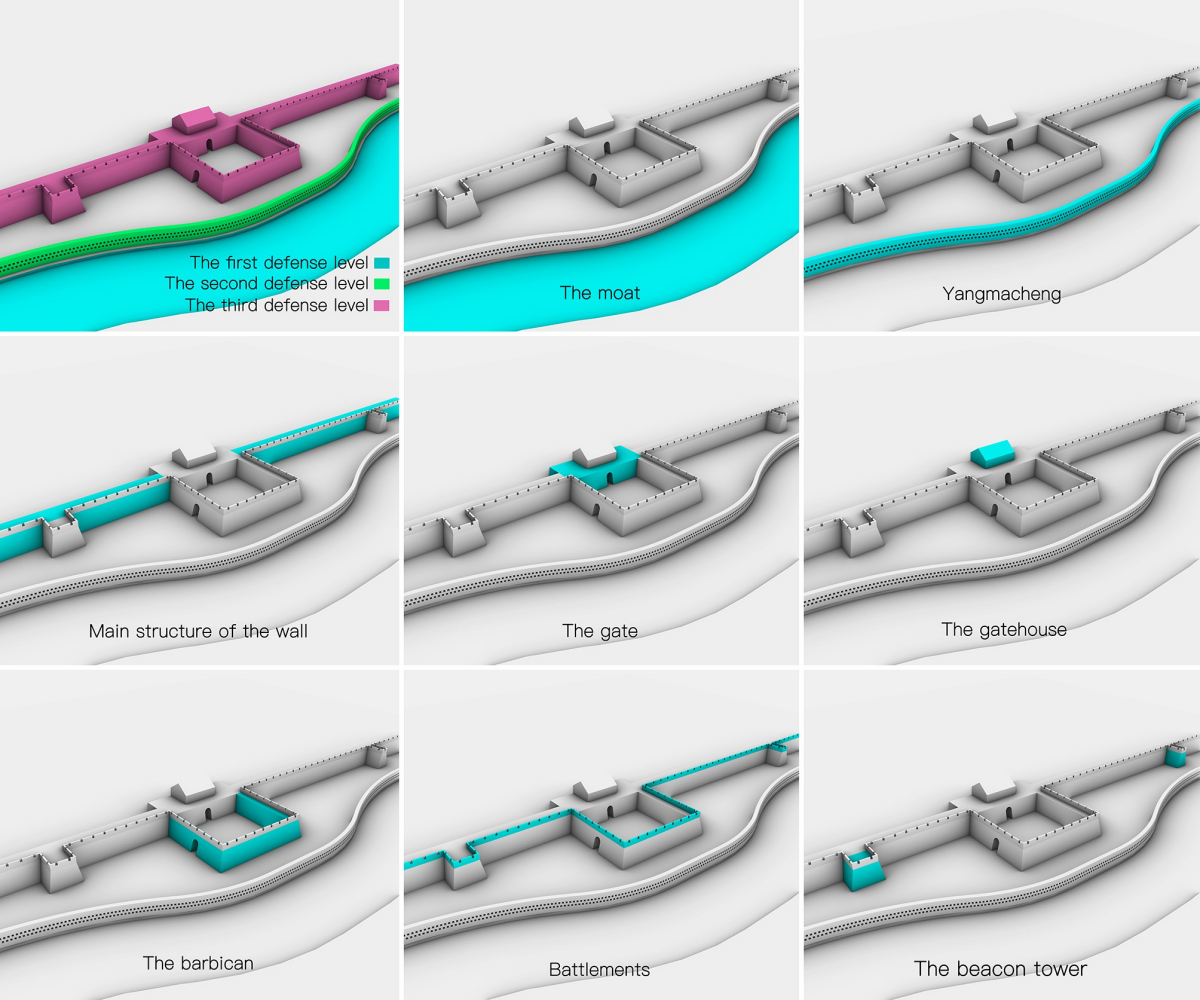
The structure of the three defense levels and the components of the third defense levels.

In the battle against the enemy, the defense mechanism and ability of the outer space near the wall are critical. The outer defense system of the wall of the coastal defense forts can be summarized into three defense levels. Fig 1 shows the structure of the three defense levels and the components of the third defense level.

### The first defense level—the moat

**Fig 2:**
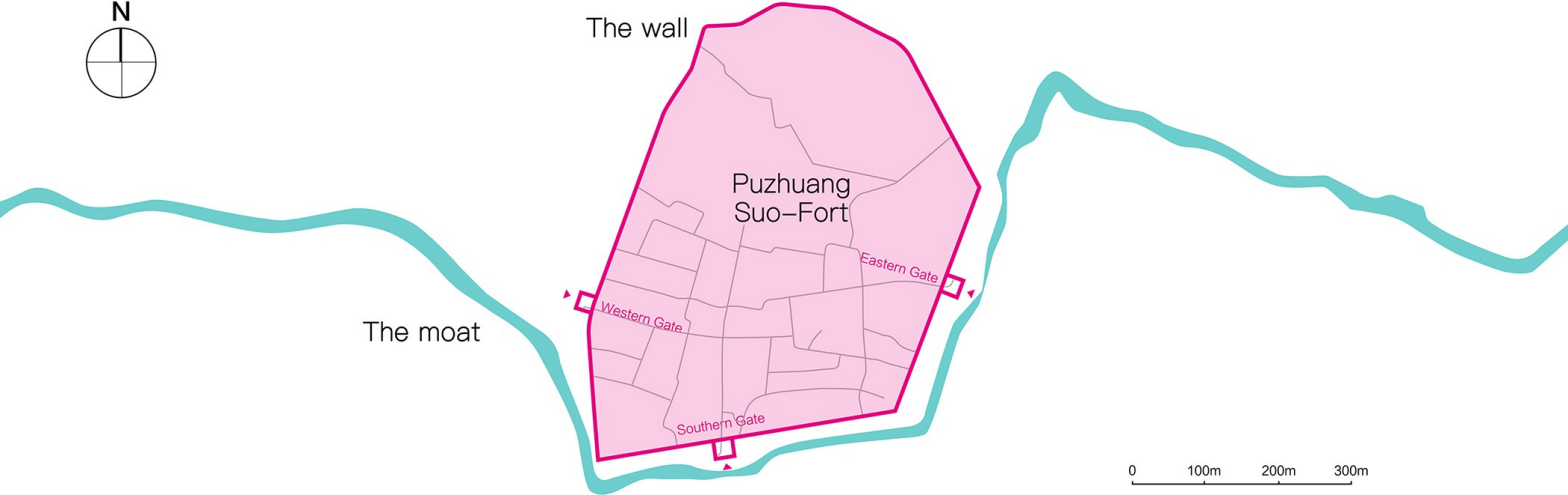
The layout and moat trend of Puzhuang Suo-Fort.

In ancient times, the moat was utilized for defense. To protect against assailants near the wall, the moat was built in front of the Wei-Forts and Suo-Forts. The moat’s design was inspired by the fort’s wall structure [14]. As depicted in Fig 2, Puzhuang Suo-Fort is partially encompassed by a moat that curves slightly near the barbican.

### The second defense level–Yangmacheng

Yangmacheng could block the enemy’s attack on the fort defense [15], where the late sheep and horses could also rest at night [16]. When facing enemies, the general guarding the fort often decided whether to send men to defend under Yangmacheng. In the Northern Song Dynasty, Zeng Gongliang wrote Wu Jing Zong Yao: "The height of Yangmacheng can be less than ten Chi (3.20 m), more than eight Chi (2.56 m) [17]." Chen Gui said in Shou Cheng Lu, "it is appropriate to build high and thick Yangmacheng, which are as high as ten Chi (3.20 m) and as thick as six Chi (1.92 m) [18]." It can be seen that the shape of the Yangmacheng after the Song Dynasty should be between eight Chi and ten Chi high (2.56–3.20 m).

### The third defense level—the fort wall

**Fig 3:**
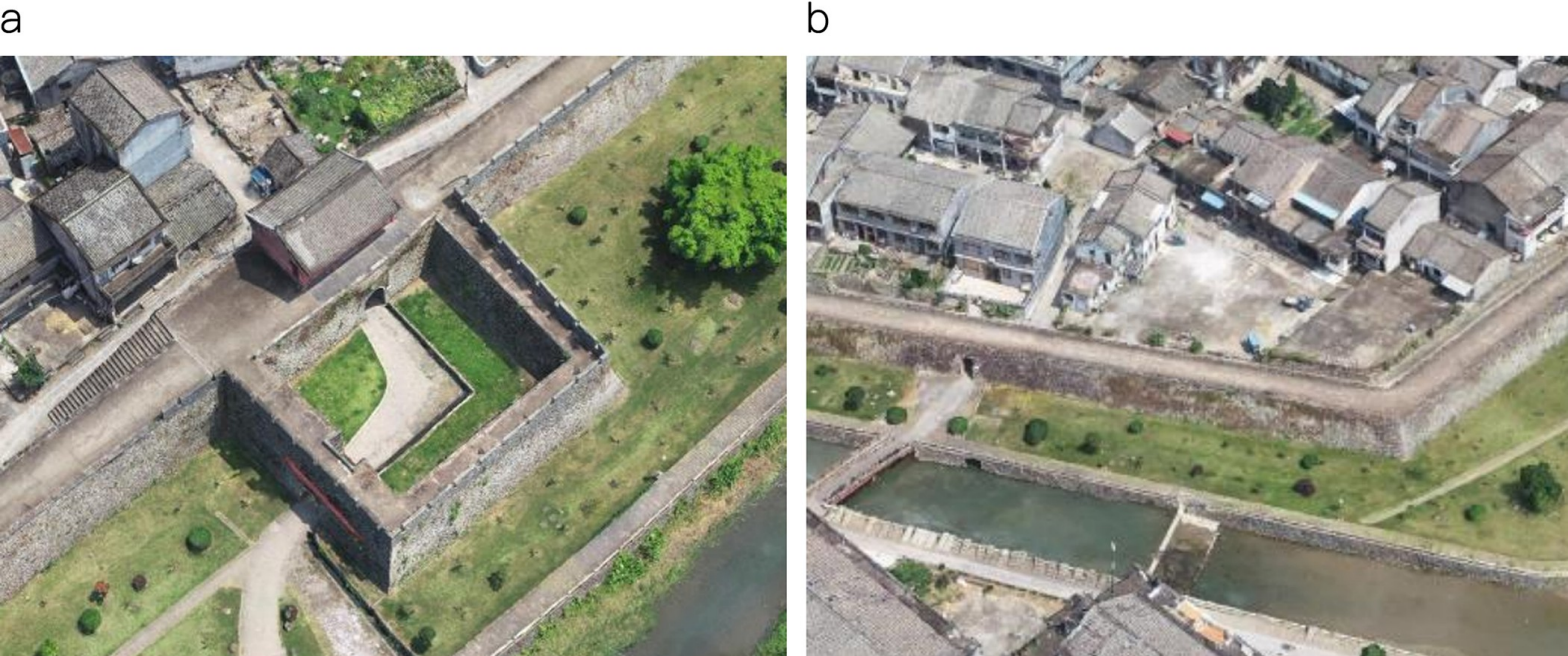
The barbican of Pu Zhuang Suo-Fort point cloud model (a) and the wall of Pu Zhuang Suo-Fort point cloud model (b).

The last line of defense of the fort was the wall, which was more complex than the first two lines [16]. In a conflict, the wall served as a passive defensive barrier. As a result, it must be formidable at the point of assault. Secondly, the wall near the gate was equipped with ancillary fortifications, such as barbicans (Fig 3), battlements, beacon towers, gate towers, turrets, and so on [5]. These ancillary structures were generally used to support soldiers’ firepower output, which is the active defense possessed by the wall. Here, introduce its components separately.

Main structure of the wall: Chen GUI’s Shou Cheng Lu proposed that the moat outside the fort, Yangmacheng, and outer space were the external fortifications [18]. The moat inside the fort and the inner space was the internal fortifications. Forts and moats became passive defense’s main forces and provided the basis for active defense.

The gate: In terms of functions, gates were divided into land and water gates. Land gates were responsible for entering the inner fort by land. Water gates were the entrance connecting the external river network to bring water into the fort. At the same time, it can also be used as the gateway to the fort’s waterway traffic [19]. The site selection for coastal defense forts in Zhejiang Province was usually set in the channel of river networks and had built moats. Water gates and moats could also be used as essential channels of flood drainage [20].

The gatehouse: gatehouse is a building built on the city gate where people can stand. In ancient times, it was used for observation and shooting at the enemy.

The barbicans: The barbican was built outside the fort. The gate of the barbican was usually set on the side [17, 21]. The moat was set up outside the barbican. Wu Jing Zong Yao contained: "The shape of a barbican is round or square, depending on the terrain. The height and thickness of barbican walls are the same as fort walls, and only one gate was opened on the side, either on the left or the right [17]". Once the enemy enters the barbican, the defenders can shoot arrows and throw stones at the enemy from the surrounding walls to put them in a dilemma.

The battlements: Battlements were small walls arranged at successive intervals on the fort wall [22]. The battlements might shield the wall from an arrow attack from underneath. Moreover, a battlement gap was established between two battlements Afterwards, staring down, one may release arrows into the gap [23]. Chen Gui proposed that old battlements were not high enough to cover the defenders. At the same time, it was defenseless against the cannon attack. It should be replaced by building a magpie platform on the wall. On top of it, a flat-headed wall should be built. The magpie platform was two Chi (0.64 m) high and five Chi (1.60 m) wide. The wall was six Chi high (1.92 m) and two Chi (0.64 m) thick. The upper and lower rows of staggered square holes were set on the wall, shaped like the character "品." The defenders could use the holes to attack climbing enemies with knives and guns [17].

The beacon tower: The beacon tower was half protruding outside the main wall. It was set up to facilitate the soldiers to observe the situation at the bottom of the wall. To a certain extent, it also solved the problem of having a dead angle for firing [24]. Chen Gui proposed that high and thick walls should be built on the beacon tower, leaving a hole in the character "品." The hole made it easy for soldiers to observe the situation below the wall and allowed knives and guns to pass through. Two small doors were opened on the left and right sides of the beacon tower. One was to observe the situation under the wall. The other was to use defensive equipment against wall climbers. Roofed huts were built in the beacon tower to shelter the guards from the wind and rain. When attacked, they were torn down, and high fork logs were erected against the walls to block the enemy [17].

### The summary of the range of weapons

Above the walls, the battlements established a dense and orderly array. Its primary function was to shape a dense firepower network with weapons to effectively block the siege. As demonstrated in Table 1, historical writings such as Wu Bei Zhi [25] and Fang Shou Ji Cheng [26] document the variety of weapons.

**Table 1:** Typical weapons and firing ranges of ming dynasty (source: Compiled and converted according to the Wu Bei Zhi and Fang Shou Ji Cheng).

Typical Weapons and Firing Ranges of Ming Dynasty (1 Bu ≈ 1.6 m, 1 Li ≈ 590 m)			
Weapon types		Firing range	Modern unit conversion
	Arrow	Within 75 Bu	Within 120 meters
	Crossbow	About 220 Bu	About 352 meters
Cold arms	Single shoot trebuchet	50 Bu away	80 meters away
	Double shoot trebuchet	60 to 80 Bu away	96 to 128 meters away
	Five shoot trebuchet	50 Bu away	80 meters away
	Seven shoot trebuchet	50 Bu away	80 meters away
	Tornado trebuchet	50 Bu away	80 meters away
	Tiger squatted trebuchet	50 Bu away	80 meters away
Firearms	Weiyuan Cannon	Within 20 Li	Within 11.8 kilometers
	Folangji Cannon	More than 1 Li	More than 590 meters
	Triple shoot blunderbuss	120 Bu away	120 meters away
	Halberd	200 Bu away	320 meters away
	Blunderbuss stick	200 Bu away	320 meters away
	Musket	Within 300 Bu	Within 480 meters

From the chart, we can summarize that the range of cold weapons is about 80 to 120 meters, and firearms can reach hundreds of meters. In the defensive war, the most commonly used arrow range of soldiers on the wall is within 120m. The distance from the moat to the city is less than 120m. Therefore, the three defense levels mentioned in this article are within the effective range of the arrows shot on the wall. Once the attack distance is no longer the limiting factor, firing network density is a measure of the defensive capacity of the outer space of the walls.

### Data modeling

The internal clues between coastal defense fortifications are gradually found after combining ancient forts’ attack and defense modes. The number and spacing of battlements on the wall are directly related to the spatial firepower network in individual combat. Battlements had a basic modulus in ancient times. Their height was generally between five and seven Chi (1.60–2.24m). Except for battlements, the wall’s height and the moat’s width were not fixed values. However, they maintained a particular range. Previous studies on them are qualitative. For instance, more labor and money should be invested in order to build a larger artificial moat. Nevertheless, if the moat is not broad enough, the defense potential is limited. The majority of forts were constructed in a methodical manner using fundamental guidelines [27].

**Fig 4:**
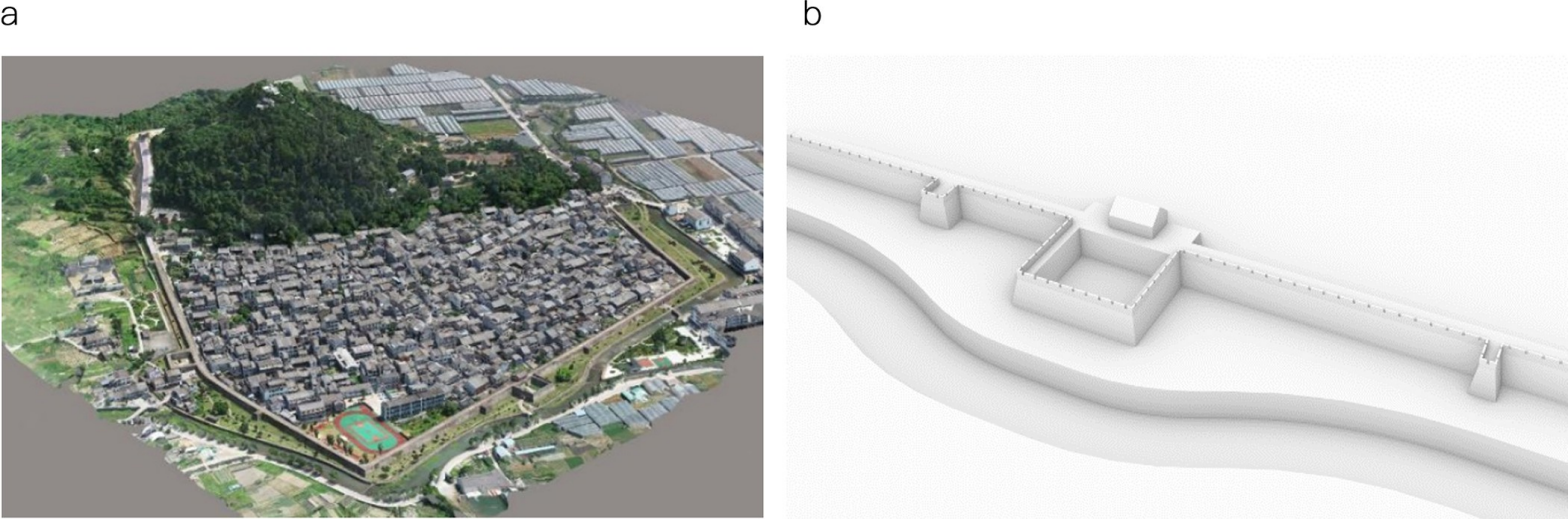
The 3D point cloud model of Puzhuang Suo-Fort (a) and the model of the south wall of Puzhuang Suo-Fort (b).

**Fig 5:**
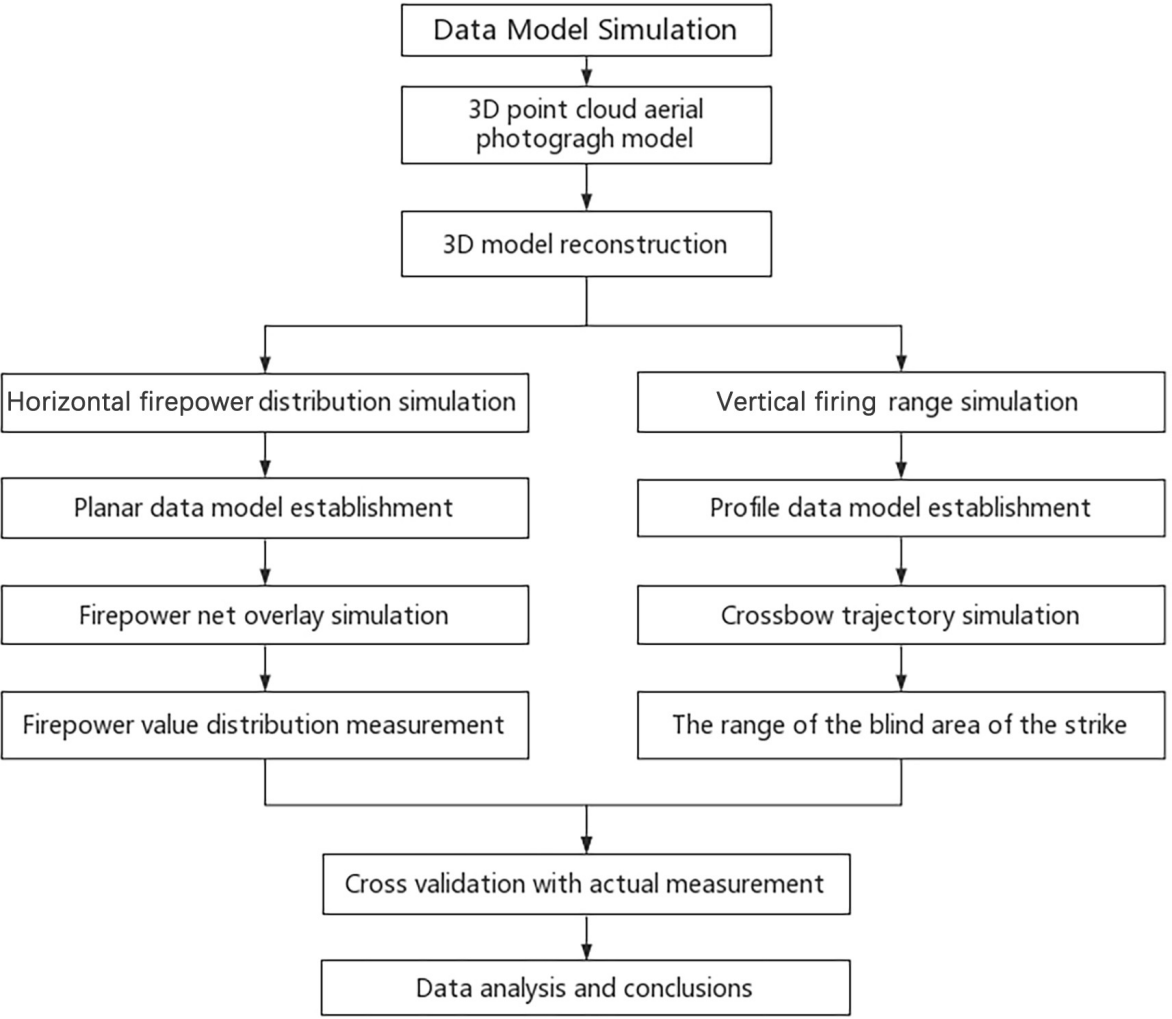
Research method and process.

According to the point cloud model in Fig 4(A), accurate data are obtained. Then the wall 3D model of Puzhuang Suo-Fort is reconstructed in Rhinoceros software, as shown in Fig 4(B). Based on this 3D model, the horizontal firepower distribution and vertical firing range can be simulated, respectively, as displayed in Fig 5.

## Calculation and results

### Horizontal firepower distribution law

Battlements are single-soldier firepower output platforms. The size of its opening directly affects the output angle and coverage area of the unidirectional firepower.

**Fig 6:**
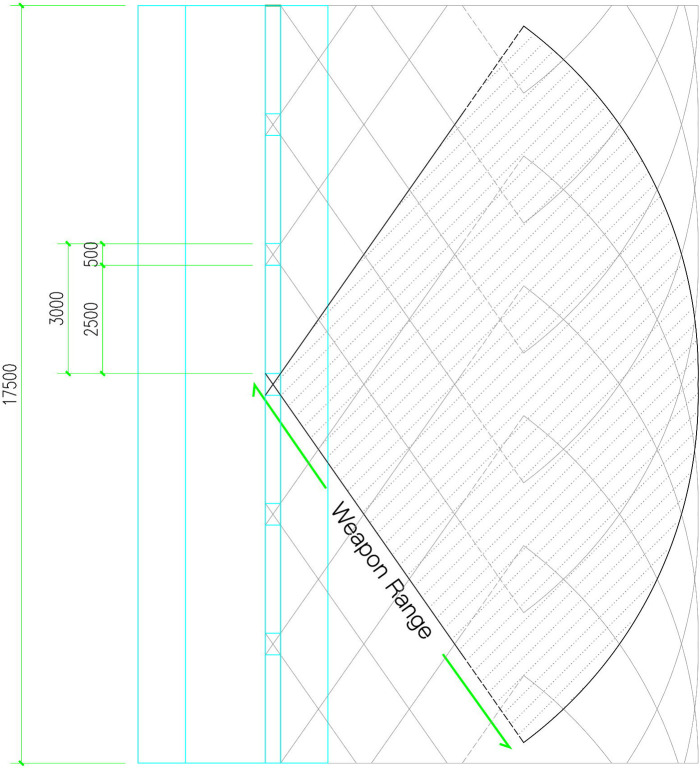
Fan-shaped firing range of a single battlement gap.

The firepower network associated with battlements and firearms is graphically expressed, taking the model of Puzhuang Suo-Fort as a reference. In the mode of long-distance attack by a single soldier, the attack range of a single battlement gap is a fan-shaped range (Fig 6) with the center of the inner battlement as the origin and the maximum range of the weapon as the diameter.

**Fig 7:**
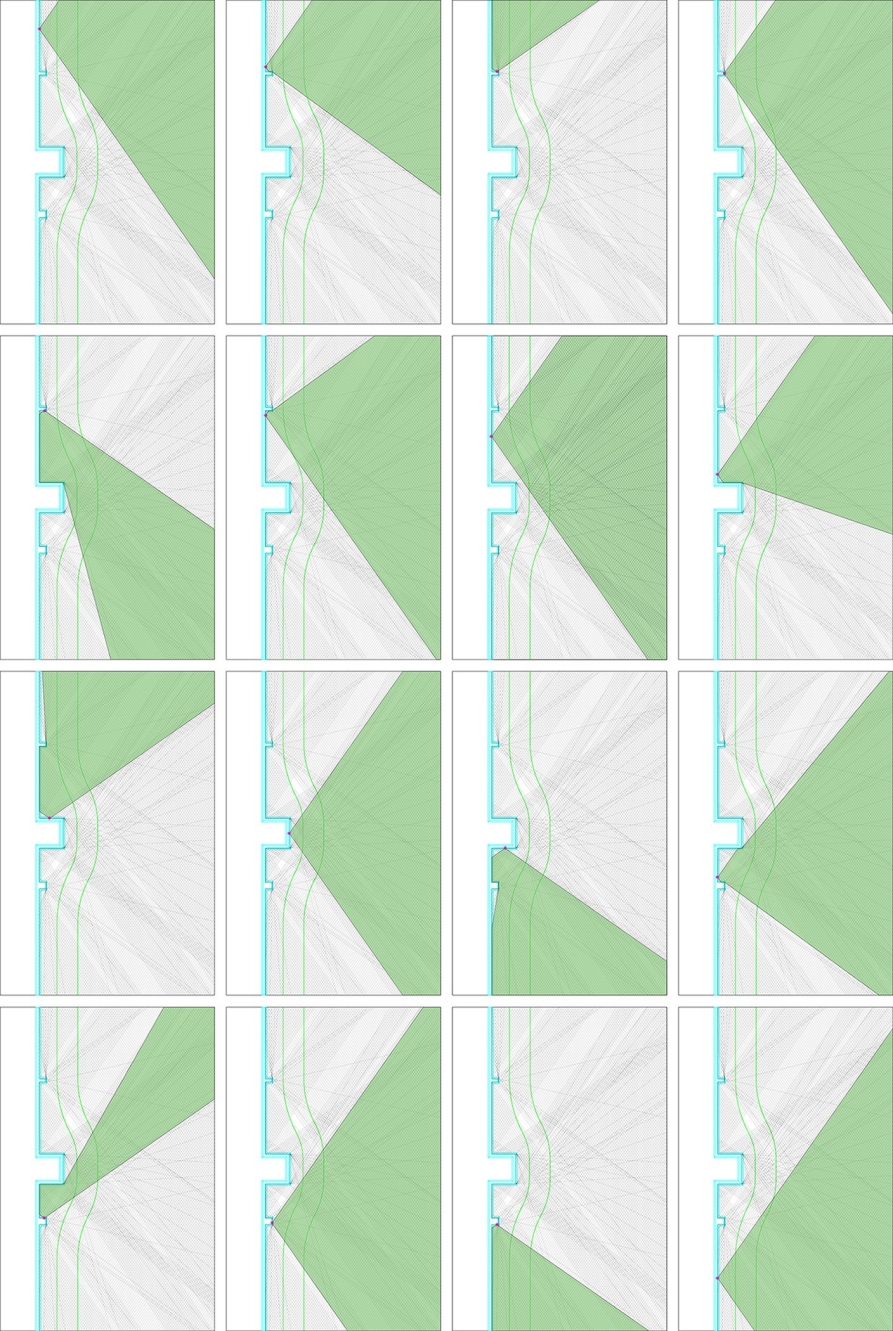
The firing range at different points of the wall.

The walls of Puzhuang Suo-Fort are not straight and continuous. The fan-shaped striking range is substituted into the south wall model of Puzhuang Suo-Fort. An area 100m away outside the wall is taken as the research range. Then take several points at different positions on the wall as an example to illustrate the firing range. Fig 7 depicts the stark horizontal surfaces of 16 sample points. It exemplifies how the placement of protruding structures, such as barbicans and beacon towers, will create different degrees of occlusion to the firing range of the battlements on its side and adjacent wall. The battlements, nevertheless, can shoot to the side, complementing the blind area beneath the wall. It differs from a single wall in that it can only shoot forward, extending the defense potential of the entire wall by some amount.

**Fig 8:**
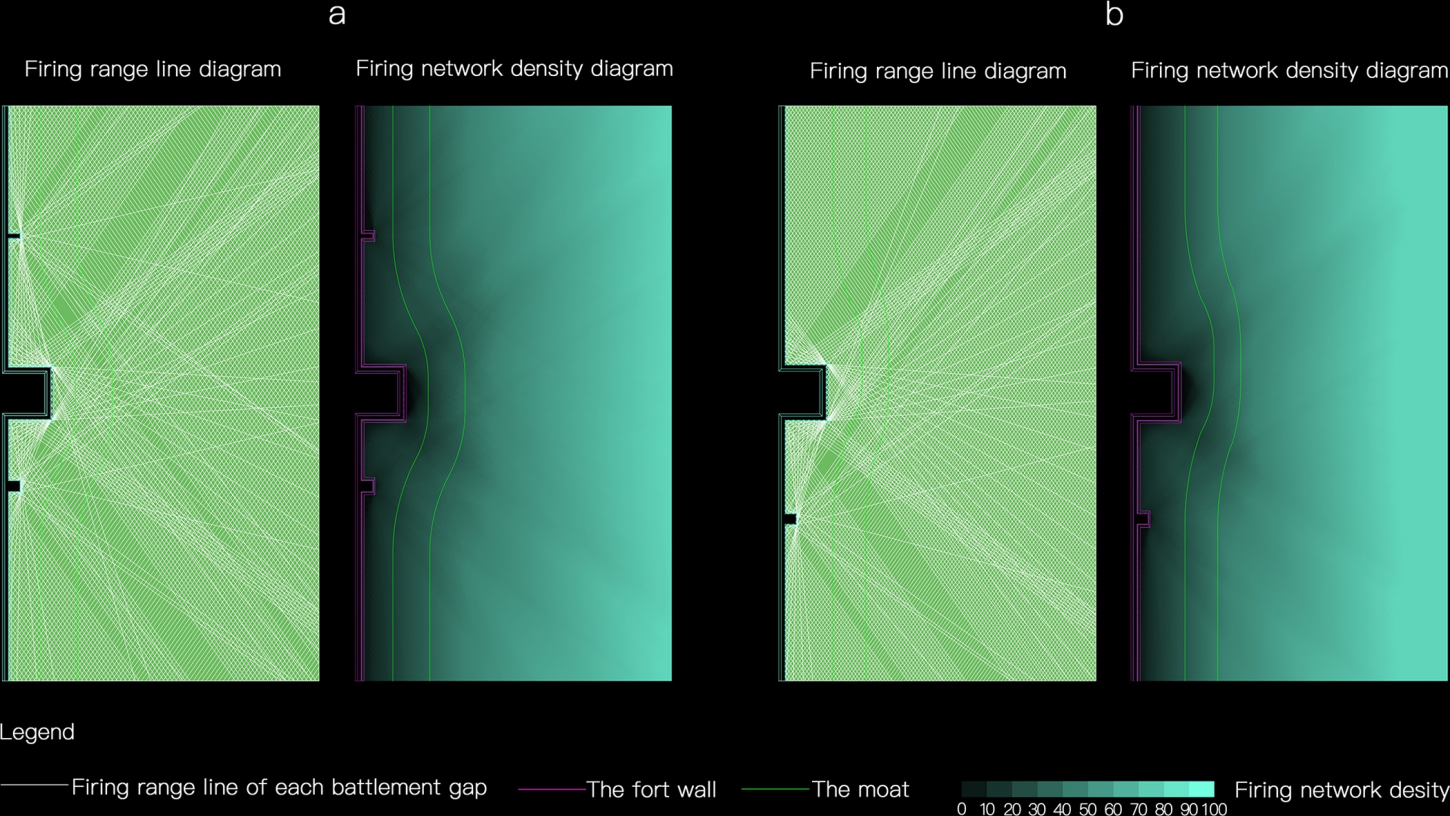
The simulation of firepower network of Puzhuang Suo-Fort south wall (a) and The simulation of firepower network of Puzhuang Suo-Fort east wall (b).

Based on this, the firing range of battlements on the southern wall(with a barbican and two beacon towers) and the eastern wall (with a barbican and a beacon tower) of Puzhuang Suo-Fort can be further superimposed in ArcGis to obtain the firepower network image of the whole wall. Set the color transparency of each firepower surface to 99%. The higher the density of firepower surfaces at a point, the darker the color overlay will be here. As depicted in Fig 8, the color depth of each point can directly reflect its firepower value. The intrinsic significance of the fort can then be evaluated qualitatively. The density of the firepower network steadily rises from near to far in the region beneath the wall. There are also minor zones of diminished firepower value in front of barbicans and beacon towers. The moat’s excavation position is within the apparent attenuation range, contributing to the wall’s defense capabilities.

It is necessary to quantify the firepower defense force of the fort in order to quantify the construction logic of fortifications more accurately. Specific quantitative methods are as follows. In GIS, we further carry out "segmentation by attribute," " element transformation," and "spatial connection analysis" on the superimposed image. Each block’s amount of overlapping firepower surfaces can be calculated. The number denotes the number of battlements capable of striking this point. The more significant the number, the greater this point’s firepower value. The firepower values of the space beneath Puzhuang Suo-south Fort’s south and east walls are then estimated and published. The firepower value of each point in the graphic may be quantitatively examined using the data in the Tables 2 and 3.

**Table 2:** Calculation of the firepower value of the space under the south wall of Puzhuang Suo-Fort.

Distance to the wall/m	0.00	2.03	3.66	4.06	4.29	6.09	7.58	8.12	8.90	9.87	10.20	11.02
Firepower surface/unit	0	1	3	4	6	7	9	10	12	13	15	16
Distance to the wall/m	12.30	12.49	14.41	15.61	16.50	17.05	17.71	18.60	20.21	20.89	22.31	22.80
Firepower surface/unit	18	19	22	24	25	26	28	29	30	33	34	35
Distance to the wall/m	24.27	24.41	24.90	26.51	28.61	29.10	30.71	31.20	32.81	34.91	37.01	38.15
Firepower surface/unit	36	37	38	39	40	43	45	46	47	48	49	50
Distance to the wall/m	38.71	39.11	41.21	43.31	44.19	45.41	47.51	48.59	49.61	50.88	51.71	53.81
Firepower surface/unit	52	53	54	55	57	56	58	60	61	63	64	66
Distance to the wall/m	55.91	58.01	60.11	62.21	64.31	65.27	66.41	68.51	70.61	72.71	74.81	76.91
Firepower surface/unit	68	70	72	74	76	78	80	82	85	86	88	90
Distance to the wall/m	79.01	81.11	83.21	85.31	87.41	88.33	89.51	91.33	93.71	95.81	97.91	100.0
Firepower surface/unit	92	94	96	98	100	102	101	103	105	107	109	111

**Table 3:** Calculation of the firepower value of the space under the east wall of Puzhuang Suo-Fort.

Distance to the wall/m	0.00	1.75	3.50	5.25	7.00	8.75	9.82	10.73	11.50	11.83	13.25	15.00
Firepower surface/unit	0	1	3	7	9	11	13	15	17	19	23	27
Distance to the wall/m	16.75	17.13	18.50	20.25	22.00	23.75	24.51	25.50	27.25	27.60	29.00	29.35
Firepower surface/unit	29	31	33	37	39	40	41	43	44	45	46	47
Distance to the wall/m	30.75	31.27	32.50	32.99	34.25	34.74	36.00	36.49	37.75	38.24	39.50	39.99
Firepower surface/unit	48	50	52	53	54	55	56	57	58	59	60	61
Distance to the wall/m	41.25	41.74	42.04	43.00	43.22	43.49	44.75	45.24	46.50	46.99	48.25	48.74
Firepower surface/unit	62	63	64	65	67	68	69	70	71	72	73	74
Distance to the wall/m	50.00	50.49	51.75	52.24	53.50	53.99	55.25	55.74	57.00	57.49	58.75	59.24
Firepower surface/unit	75	76	77	78	79	80	81	82	83	84	85	86
Distance to the wall/m	60.50	60.99	62.25	62.74	64.00	64.49	65.75	66.24	67.50	68.10	69.25	70.13
Firepower surface/unit	87	88	89	90	91	92	92	93	94	96	95	98
Distance to the wall/m	71.00	71.49	77.75	73.24	74.50	74.99	76.25	76.74	77.35	78.00	78.49	79.75
Firepower surface/unit	99	100	101	102	103	104	105	106	107	108	109	110
Distance to the wall/m	80.27	81.50	81.90	83.25	83.74	85.00	85.49	86.75	87.24	88.50	88.99	90.25
Firepower surface/units	111	112	113	114	115	116	117	118	119	120	121	122
Distance to the wall/m	90.74	92.00	92.49	93.75	94.24	95.50	95.99	97.25	97.74	99.00	99.49	100.8
Firepower surface/unit	123	124	125	126	127	128	129	130	131	132	133	134

**Fig 9:**
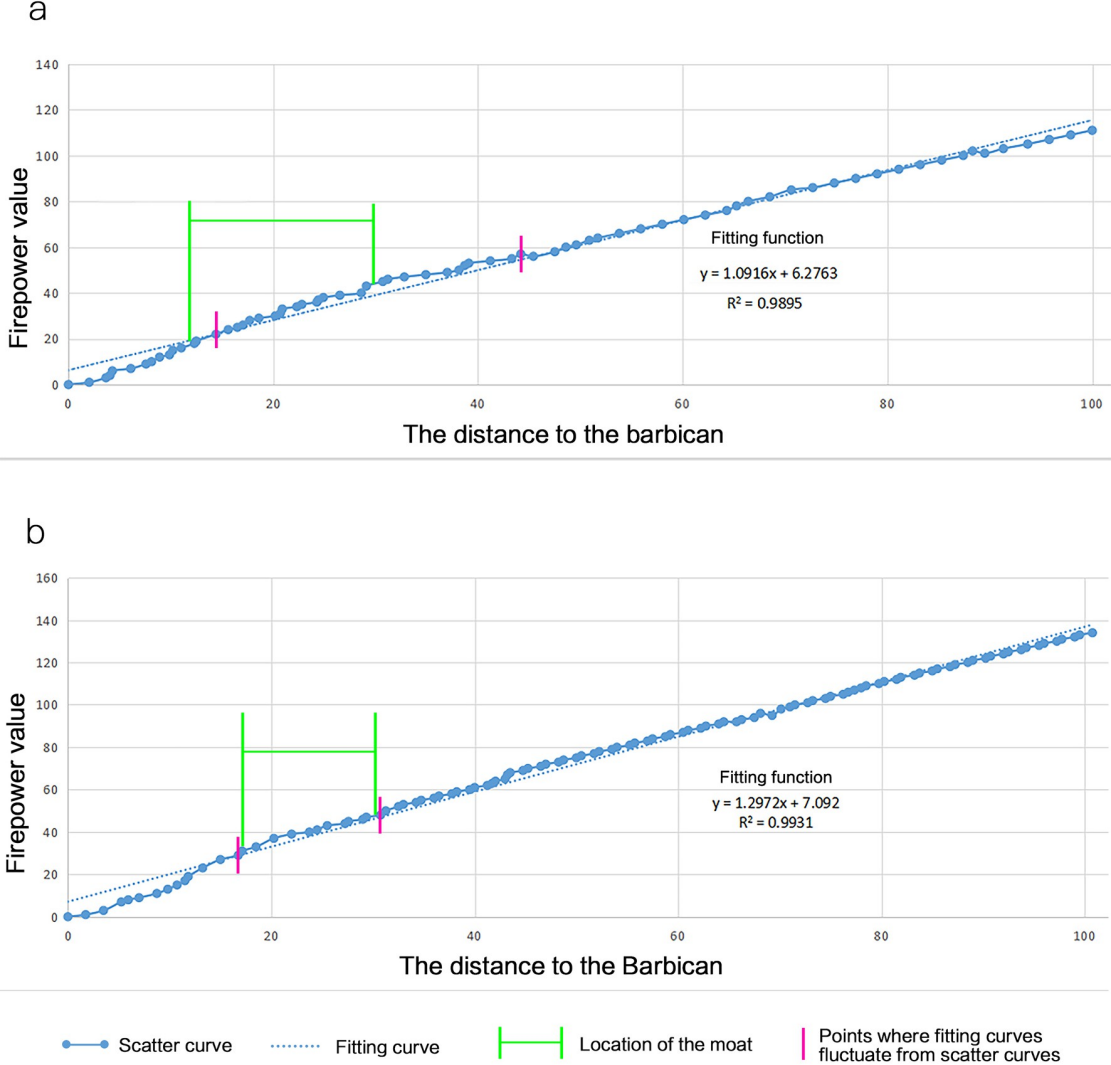
Scattered chart of firepower values in the space under the south wall of the Puzhuang Suo-Fort (a) and scatter diagram of space firepower value under the east wall of Puzhuang Suo-Fort (b).

In GIS, the area of 100m in front of the barbican in the overall wall model is drawn. And the firepower value of the block segmented by the range line is calculated respectively. We set the distance from a point outside the barbican to the barbican as the independent variable and the firepower value at that point as the dependent variable. In turn, scatter plots can be drawn. A fitted line and R^2^ can be calculated by the command "trend line" in Excel based on the scatter plot. R^2^ is the coefficient of determination. It indicates the proportion of the sum of squares to the sum of squares of the total errors and reflects the degree of fit of the regression line. Its value ranges from 0 to 1. The more R^2^ is close to 1, the better the regression equation is fitted. The more R^2^ is close to 0, the worse the regression equation is fitted. According to the image of space firepower value under the south wall of Puzhuang Suo-Fort in Fig 9(A), it can be seen that its function image can be approximately fitted to a straight line: Y = 1.0916x + 6.2763, R^2^ = 0.9895. There is a large deviation between the image and the fitted line at 0–43.3m away from the fort wall, indicating that the firepower value has a nonlinear and rapid decay in this range. Moreover, the firepower value depicted in the image drops dramatically at 0–16.5m from the fort wall. It is lower than the fitted line, implying that decay of firepower is faster in this area. Note the location of the moat in the picture. The position of the moat is set between 11.9m and 29.9m from the fort, which is definitely in the area where firepower attenuation occurs. Furthermore, the huge attenuation line of 16.5m from the fort is located in the plane range of the moat, signifying that the moat’s location is scientific to a certain extent and may boost defense ability in the area of firepower attenuation.

Similarly, the function in the calculation diagram of firepower value under the east wall of Puzhuang Suo-Fort in Fig 9(B) is also a fitting line: Y = 1.2972x + 7.092, R^2^ = 0.9931. There is a large deviation between the image and the fitted line at 0–32.5m away from the fort wall, indicating that the firepower value has a nonlinear and rapid decay in this range. Moreover, at 0–16.8m away from the fort wall, the image drops significantly. It is lower than the fitted line, indicating that the fire attenuation is more rapid in this area. The location of the moat is marked in the figure, which is set within 17m-30.3m of the fort. Compared with the south fort wall, the moat of the east fort wall is set closer to the decay limit.

In a nutshell, the attenuation of firepower value on the moat surface exhibits an uneven, somewhat rapid oscillation. That is, the placement of the moat plays a crucial defensive role in areas with limited firepower. Yangmacheng is typically placed 10m beyond the wall, adding to the fort’s defensive capability.

**Fig 10:**
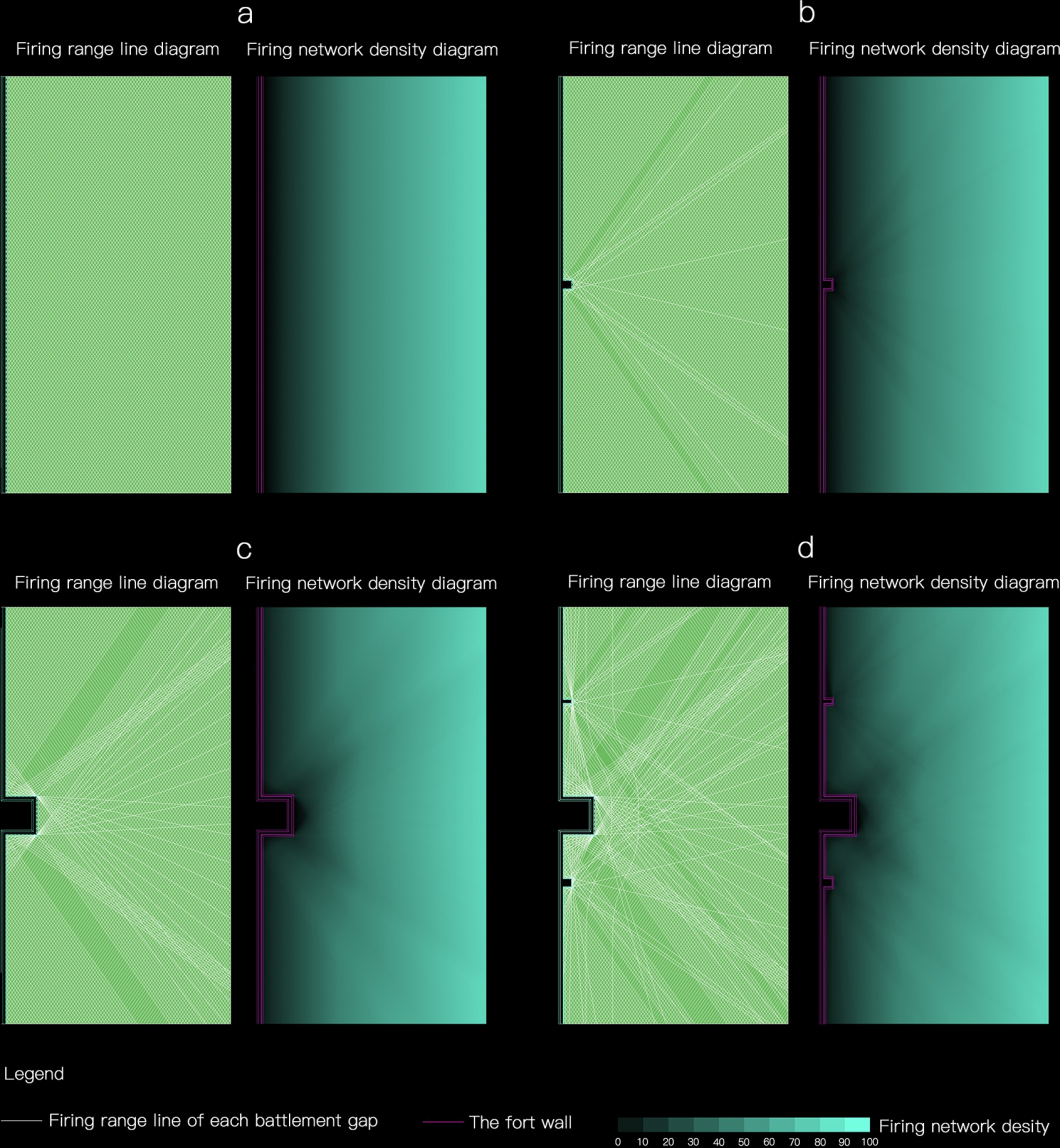
Model of the firepower value distribution of the walls: a-The single wall; b- The wall with beacon towers; c-The wall with barbicans; d-The overall wall.

In order to avoid the influence of the intera0ction of different wall fortifications, we take the south wall fortifications as an example to further study the firepower value distribution of the single wall, the wall with beacon towers, the wall with barbicans, and the overall wall in Fig 10. As can be observed in Fig 10(A), the fire coverage outside the fort wall is extremely uniform due to the uniform and equidistant distribution of battlements above the fort wall. The closer it approaches the wall, the more linear the diminishing law becomes. The firepower values in Fig 10(B) and 10(C) diminish radially from outside to inside, with barbicans or beacon towers in the center of the circle. The greater the outer convex fortification volume, the wider the attenuation range. Additionally, the two angles of the outer convex attenuation are the most prominent, and the attenuation ray extends to a considerable distance. In the overall wall model in Fig 10(D), the attenuation area is the most disordered. It is the superposition result of the radial decline of several fortifications.

Through the quantitative firepower value measurement mentioned above, the firepower coverage model of the barbicans, the beacon towers, single and overall walls are measured about the distance to the fort. The interval distance is set every 5 meters, and 100m is taken as the research range. The distribution of firepower values for each of the four scenarios is presented in Table 4.

**Table 4:** Measurement table of fortifications on firepower value.

Distance to the fort/m	5	10	15	20	25	30	35	40	45	50	55	60	65	70	75	80	85	90	95	100
The single wall	5	9	15	19	23	29	33	37	43	47	53	57	61	67	71	77	81	85	91	95
The wall with beacon towers	8	14	20	24	30	34	38	44	48	54	58	62	68	72	78	82	86	92	96	100
The wall with barbicans	5	11	23	29	40	44	48	56	60	64	70	74	80	86	90	96	100	104	110	114
Overall wall	7	15	24	30	39	45	49	54	56	63	68	72	78	85	90	94	98	103	107	111

**Fig 11:**
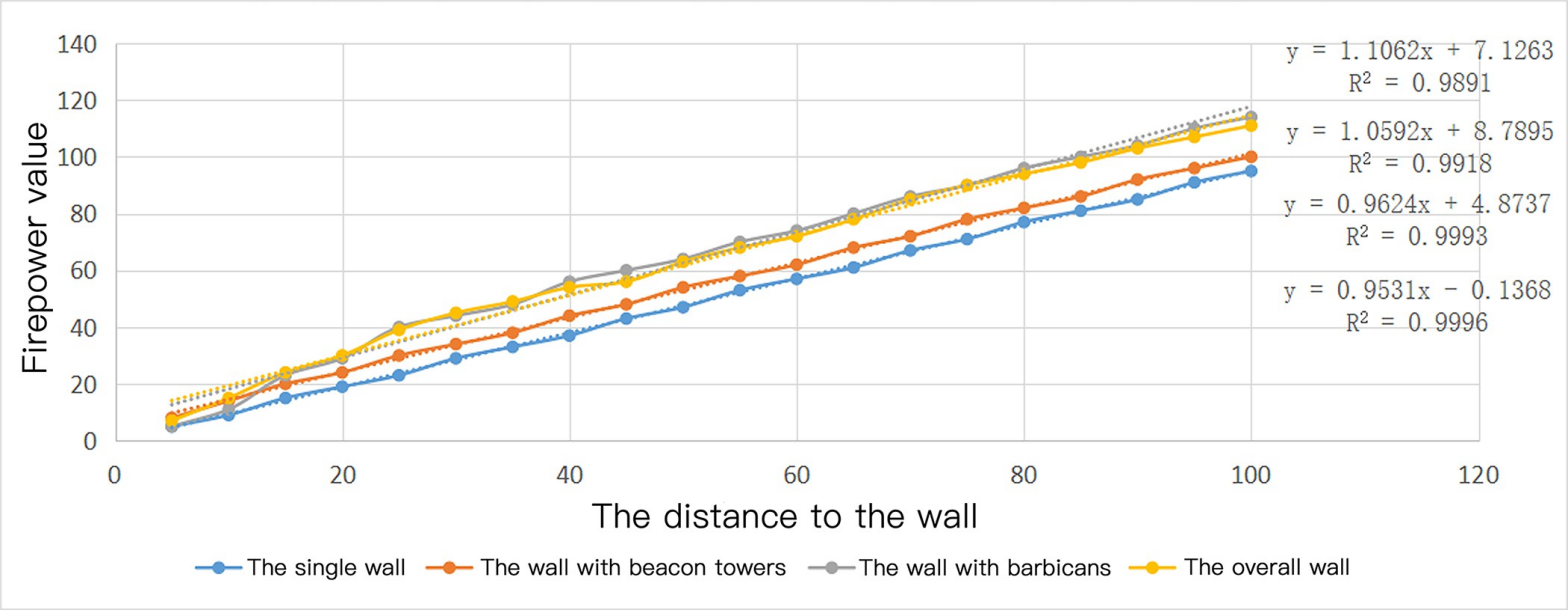
Measurement graph of walls on firepower value.

Scatter linear analysis was conducted on the data to obtain Fig 11. Under the four simulation conditions, the lateral distribution of the firepower value can be fitted into a linear relation. The R^2^ order of the fitting line is: the single wall > the wall with beacon towers > the overall wall > the wall with barbicans. Consequently, the placement of fortifications atop the wall will render the distribution of firepower on the plane increasingly complicated and disordered. The order of defense ability can be seen as: the overall wall ≈ the wall with barbicans > the wall with beacon towers > the single wall. So the following conclusions can be drawn. If defenses protrude on the walls (such as the barbicans or the beacon towers, etc.) in the relatively far part of the wall, a power attenuation area will be formed. Nevertheless, owing to the enhanced firepower on all three surfaces, the original weak defense underneath the wall may be upgraded. The original layered firepower coverage’s attenuation may be transformed into radial attenuation with convex fortifications at the center of the circle. It outperforms the original single-wall defense’s performance

The following can be obtained from the cross-validation of the scatter plot of firepower value and the actual measured value. In fort spatial defense, the attenuation law of horizontal firepower network is highly correlated with the spatial position of actual fortifications. The moat serves as the first intercept for the coverage attenuation of the wall’s firepower value, while the space under Yangmacheng serves as the second defense line for the coverage attenuation of the firepower value. The setting of the two lines of defense is basically at the "inflection point" of the attenuation mutation. The interception of fortifications like this can carry out the most effective defense reinforcement at the point where the enemy launches a charge and realize efficient and accurate defense.

### Vertical firing range simulation law

The battlements of the wall mentioned above also have basic regulatory information on the defense fortifications of Yangmacheng under the fort. The wall height is about 8 to 10 Chi high (2.56–3.20 m), and it is about 10 meters away from the fort [17]. Meanwhile, according to the point cloud model, it is determined that the width of the moat is 18 meters. The height of battlements is 1 m. The width of battlements is about 350–500 mm. And the batter of the wall is about 20%.

When integrated with the horizontal firepower distribution analysis presented above, it is apparent that the moat represents the initial fire attenuation barrier. In addition, the moat can decrease the effectiveness of an enemy siege in front of the fort. Simultaneously, the firepower output on the wall and Yangmacheng beneath the fort may provide cross-firepower coverage to round out the formidable resistance in the space under the wall. "If Yangmacheng is guarded, weapons can be deployed on both sides," Chen Gui remarked in Shou Cheng Lu [18]. It can be noticed that soldiers can be stationed at Yangmacheng to confront pirates with short weapons. The spear was a short weapon significantly expanded shooting range in the Ming dynasty. The Ming Dynasty hero against the pirates, Qi Jiguang, skillfully used the Langxian to solve the problem that the Ming army was not good at close combat [28]. The maximal length of Langxian is sixteen Chi (5.12 m) [29].

**Fig 12:**
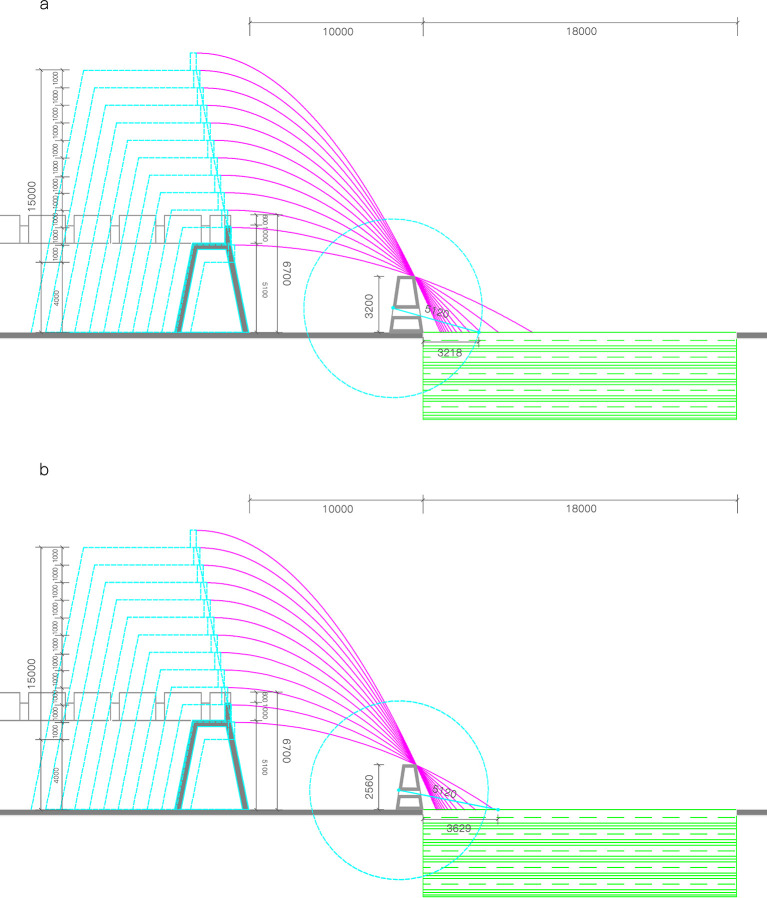
Wall Height and Moat Firepower Coverage model: a—Ten Chi (3.20 m) high Yangmacheng, b—Eight Chi (2.56 m) high Yangmacheng.

As shown in Fig 12, the effective firing of the moat can be divided into two sources. One is firepower output from holes in Yangmacheng. Its effective firing width on the moat is between 3.217897 m and 3.629440 m according to the figure. The second is the top-down firepower output of battlements. However, the actual height of the wall affects the effective range of battlement gap firepower output over the moat. If the arrow angle is controlled as flat throws, the arrow’s parabolic trajectory can be simulated according to the point that the soldier needs to hit on the wall. The higher the wall, the wider the range. However, increasing the wall infinitely would bring problems such as erosion and collapse after soaking in the rain [11]. The masonry cost would be exorbitant if the walls were excessively high. As a result, with the firepower network under Yangmacheng, the firepower network from the wall’s battlements should at least comply with the requirement of covering the whole moat, saving the most costs and enhancing strike efficiency.

**Fig 13:**
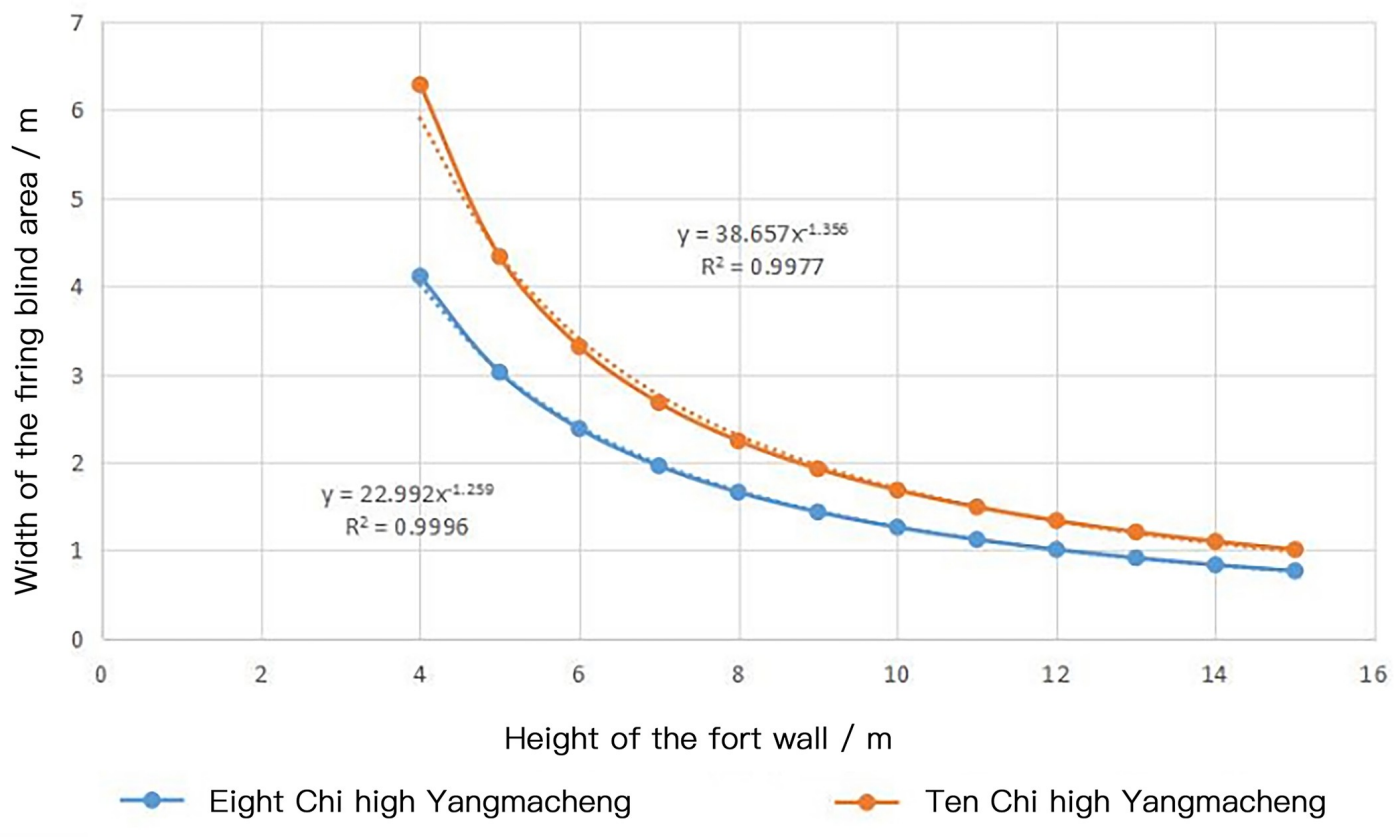
Graph of firing blind area length under different wall heights.

According to the actual measurement, the height of the south wall of Puzhuang Suo-Fort is about 5100mm. The battlement is about 1000mm higher than the wall. The distance between the moat and the wall is taken as the fixed value. The height of the wall is between 2.56 m and 3.20 m. The critical condition is drawn respectively. It can be obtained that when the height of Yangmacheng is ten Chi (3.20 m), the maximum hitting width of the subsequent spear on the river surface is 3.217897m. When the height of Yangmacheng is eight Chi (2.56 m), the maximum strike width of the subsequent spear on the river is 3.629440 meters. The height of walls in Wei-Forts and Suo-Forts recorded in ancient books is roughly between 4m and 15m. Hence, it is selected as the study range, and every 1m is utilized as a counting point for firepower output to simulate the blind area range of the battlements on the wall at various heights. Then Table 5 and Fig 13 are obtained.

**Table 5:** Firing blind area length under different wall heights.

The height of the wall/m	4	5	6	7	8	9	10	11	12	13	14	15
Firing blind area length of Eight-Chi high Yangmacheng/m	4.11	3.02	2.38	1.96	1.66	1.44	1.26	1.12	1.01	0.91	0.83	0.77
Firing blind area length of One-Zhang high Yangmacheng/m	6.29	4.34	3.31	2.68	2.24	1.93	1.68	1.49	1.34	1.21	1.10	1.01

The function images shown above can be obtained from the simulation models corresponding to the eight Chi (2.56 m) high and ten Chi (3.20 m) high Yangmacheng, respectively. The absolute value of the slope of the two curves is gradually decreasing. In other words, when the wall increases a fixed height, the firing blind area width decreases. The decreased value is not fixed but gradually decreases. The following fitting equations can be obtained by fitting the scatter plot in Excel. When Yangmacheng is eight Chi (2.56 m) high, the relationship between the wall height and the width of the firing blind area on the river is Y_1_ = 22.992x^-1.259^, and the fitting degree R^2^ is 0.9996. When Yangmacheng is ten Chi (3.20 m) high, the relationship between the wall height and the width of the striking blind area on the river surface is Y_2_ = 38.657x^-1.356^, and the fitting degree R^2^ is 0.9977. When Y_1_ = 3.629440 and Y_2_ = 3.217897 (effective firing width of Yangmacheng) are substituted into the equation, it can get X_1_ = 4.333159843, X_2_ = 6.25470017. Therefore, if the height of the wall is within the range of 4.33m-6.25m, it can meet the financial requirements based on meeting the defense conditions. The measured height of the south wall of Puzhuang Suo-Fort was 5.10m. And Pingyang County History recorded that the height of the Puzhuang Suo-Fort wall was "fifteen Chi" (4.80 m), both of which were within the calculation range.

Then, the derivative equation is used to test the relationship between economy and defensiveness. The slope value is greater than 1 (that is, when the wall is increased by 1m, the firing blind area decreases by 1m), which means that the slope value is more concerned about the economy. The slope value is less than 1, which means that the slope value is more concerned with defensiveness. And the slope value is close to 1, which means that the economy and defensiveness are balanced. The derivative equations are Y_3_ = 28.946928x^-2.259^, Y_4_ = 52.418892x^-2.356^. Then substitute x = 4.8, respectively. The slopes are Y_3_ = 0.84, Y_4_ = 1.30 and the average value is 1.07. Therefore, the external defense system of Puzhuang Suo-Fort and the construction height of the wall are a whole defense system. It also considers the overall construction economy based on satisfying the defense performance.

## Discussion

**Fig 14:**
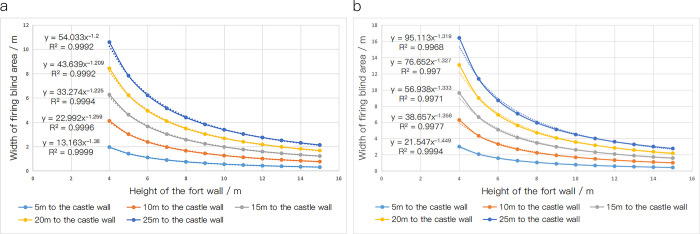
Scatter plot of the width of the firing blind area of Eight-Chi (2.56 m) Yangmacheng (a) and scatter plot of the width of the firing blind area of ten-Chi (3.20 m) Yangmacheng (b).

The above vertical firing range model verifies the science of constructing the outer defense system of Puzhuang Suo-Fort. A comparison of other fort walls shows that the height of the wall and the distance from the wall to the moat vary widely. For example, the construction height of the Palace Museum is 10m. The measured height is 9.90m. And the distance is 20m from the moat [30]. On this basis, it is further speculated that the wall height is related to the distance from the moat. In the following, the moat’s situation is stimulated and discussed: 5m, 10m, 15m, 20m, and 25m away from the fort wall. The width of the firing blind area on the moat is calculated to get Tables 6 and 7 according to the above method. After scatter plot drawing and image fitting processing, Fig 14 is obtained.

**Table 6:** Sheet of the width of the firing blind area of eight-Chi Yangmacheng.

The height of the wall /m	5m to the wall	10m to the wall	15m to the wall	20m to the wall	25m to the wall
4	1.952981	4.110461	6.26794	8.425419	10.582898
5	1.416918	3.020300	4.623682	6.227064	7.830446
6	1.102674	2.380764	3.658854	4.936944	6.215034
7	0.895472	1.958862	3.022253	4.085643	5.149034
8	0.748341	1.659169	2.569998	3.480826	4.391654
9	0.638372	1.435108	2.231844	3.028580	3.825317
10	0.55302	1.261166	1.969313	2.677460	3.385606
11	0.484829	1.122174	1.759519	2.396865	3.034210
12	0.429085	1.008536	1.587986	2.167437	2.746887
13	0.382658	0.913879	1.445100	1.976321	2.507542
14	0.343389	0.833807	1.324225	1.814644	2.305062
15	0.309737	0.765185	1.220632	1.676079	2.131526

**Table 7:** Sheet of the width of the firing blind area of ten-Chi Yangmacheng.

The height of the fort wall /m	5m to the wall	10m to the wall	15m to the wall	20m to the wall	5m to the wall
4	3.001209	6.285968	9.608468	13.066667	16.400000
5	2.068020	4.335126	6.639605	9.000000	11.357455
6	1.573379	3.312306	5.081789	6.922050	8.708259
7	1.262174	2.677140	4.114924	5.607604	7.062576
8	1.046831	2.242327	3.454063	4.708808	5.937219
9	0.888483	1.925030	2.972816	4.054298	5.117688
10	0.766980	1.682786	2.606222	3.555908	4.493618
11	0.670744	1.491517	2.317383	3.163496	4.002238
12	0.592615	1.336519	2.083758	2.846384	3.605140
13	0.527914	1.208285	1.890782	2.584728	3.277478
14	0.473450	1.100387	1.728623	2.365109	3.002454
15	0.426471	1.008315	1.590394	2.178125	2.768295

**Fig 15:**
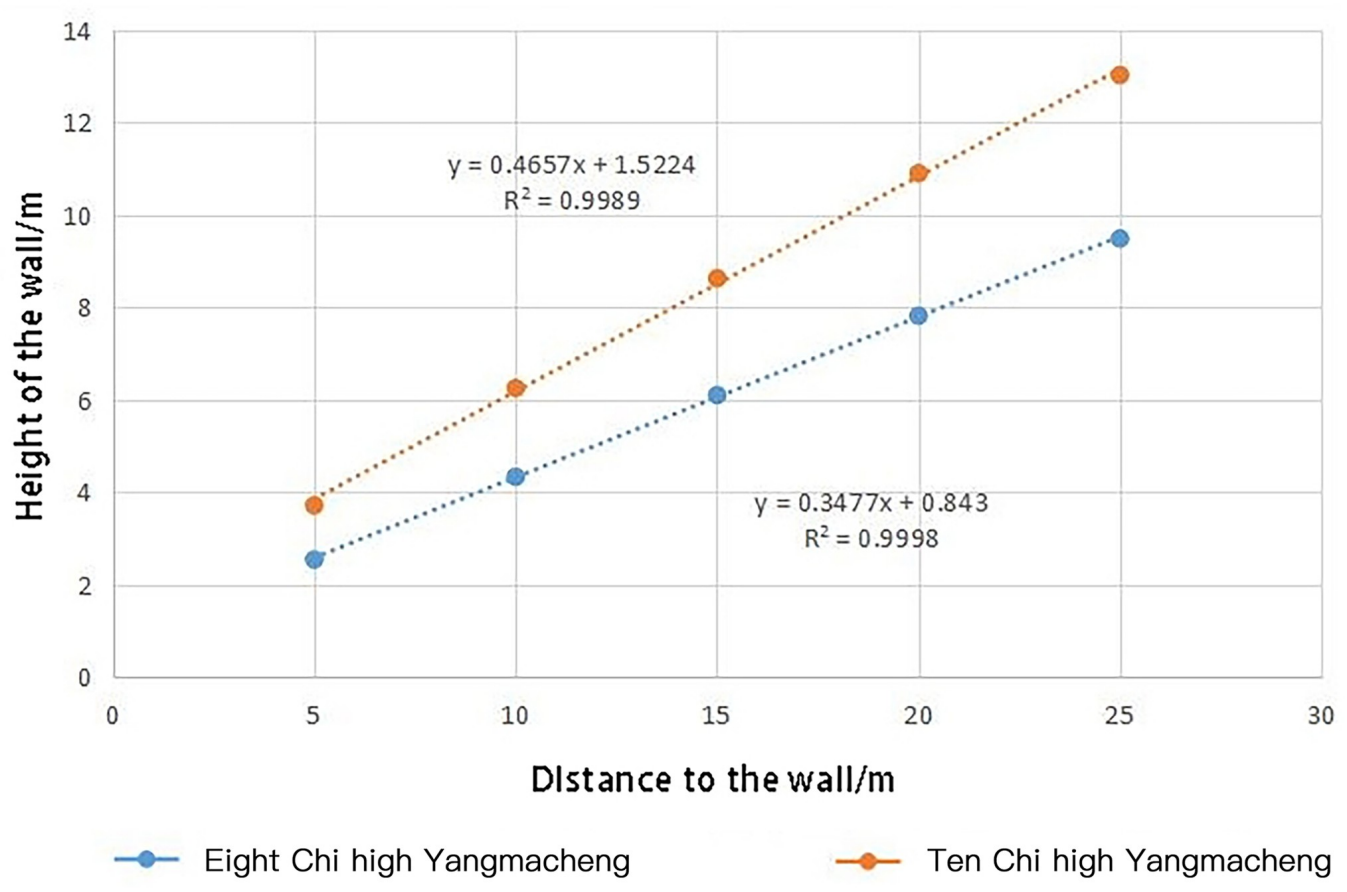
A linear relationship between wall height and the moat position.

Then, the height of the wall can be calculated as: At 2.54 m—3.71 m, 4.33 m—6.25 m, 6.10 m—8.63 m, 7.82 m—10.91 m, and 9.49m - 13.03 m, the moat with higher combat efficiency is 5 m, 10 m, 15 m, 20 m and 25 m away from the fort wall. It can be concluded that the higher the wall is, the farther the moat should be excavated. There is a linear relationship (Fig 15) between them.

## Conclusions

This study conducts a microscopic examination of a single fort’s defensive space. The defense system outside the fort is separated into three defense levels: moat, Yangmacheng, and fort wall. Additionally, it is hypothesized that the three defensive levels outside the city are intrinsically linked and comprise a systemic totality. In addition, it is connected to the weapon range, firing network density, and ancient army defensive operation style. Field research, 3D point cloud photography, and modeling were carried out on the ruins of the Puzhuang Suo-Fort in this work. ArcGIS was employed to simulate the density of the firepower network in the horizontal direction by integrating the specific data into the model. Rhinoceros and data operations were used to simulate the trajectory of weapons in the vertical direction. A three-dimensional simulation of the defense system of Puzhangosho Castle was realized.

Combined with the specific data on the engineering of Zhejiang coastal defense, this study makes a qualitative and quantitative analysis of its internal causes. This serves as a hint to determine the appropriate spatial mode of fort defense with attackers as the entry point. Using Puzhuang Suo-Fort as a typical case for partial model reconstruction and integrating it with fort engineering fire defense quantification, it is demonstrated that fort engineering design exhibits a substantial spatial connection. Besides, it illustrates the spatial regularity of fort defensive engineering design, and the following conclusions are obtained.

First, horizontally, the firepower network density of weapons outside the walls of Puzhuang Suo-Fort shows a pattern of decreasing firepower intensity from far to near within the range. The additional facilities on the walls, such as barbicans, battlements, and other outwardly convex structures, supplemented the fire intensity on the sides while causing a fan-shaped fire density decay area directly in front of them. Further, the locations of the moat and Yangmacheng were within the firepower density attenuation zone. Effectively, a protective barrier was established at the weak point of the defense.

Secondly, the height of the wall construction and the location of the moat also have linkage in the vertical direction. Soldiers under the Yangmacheng fought with weapons in hand, effectively stopping the enemy from disembarking. Yangmacheng, in the meantime, generated a blind spot for fire from the bowed soldiers on the walls. The higher the wall, the narrower the firing blind zone on the river and the relatively high construction cost. The economy and defensibility of the wall may be fulfilled when the troops’ attack range beneath Yangmacheng covers the striking blind zone on the river. The Puzhuang Suo-Fort’s data verifies this norm.

The possible errors in the model discussed in this paper are as follows: although the wall batter is mainly close to 20%, it is still not a specific value. Secondly, the height of the water level in the moat also changes over time. The model idealizes it to be flush with the bank.

The insights offered in this paper utilizing quantitative analysis can be applied to assess the defensive efficacy of ancient city sites as a promise for additional investigation. Through realistic modeling, the methods of firepower network density simulation, as well as weapon trajectory simulation can be employed to analyze and evaluate the defensive efficiency of each point of a circle of walls of a single ancient castle. In addition, it can draw comparisons between the defensive efficiency of the wall systems of several ancient coastal defense castles. It paved the way for future microscopic studies on the defensive systems of coastal fortifications.

## Supporting information

S1 File 3D model of east and south wall of Puzhuang Suo-Fort. (ZIP)

S2 File Simulation of the firepower value distribution of the walls. (ZIP)

S3 File Simulation of firing blind area. (ZIP)
